# 1-[(1-Benzyl-1*H*-1,2,3-triazol-4-yl)meth­yl]indoline-2,3-dione

**DOI:** 10.1107/S1600536814008423

**Published:** 2014-04-18

**Authors:** Fatima-Zahrae Qachchachi, Youssef Kandri Rodi, El Mokhtar Essassi, Michael Bodensteiner, Lahcen El Ammari

**Affiliations:** a Laboratoire de Chimie Organique Appliquée, Université Sidi Mohamed Ben Abdallah, Faculté des Sciences et Techniques, Route d’Immouzzer, BP 2202 Fès, Morocco; b Laboratoire de Chimie Organique Hétérocyclique, URAC 21, Pôle de compétences Pharmacochimie, Université Mohammed V-Agdal, BP 1014 Avenue Ibn Batouta, Rabat , Morocco; c X-Ray Structure Analysis, University of Regensburg, D-93053 Regensburg, Germany; d Laboratoire de Chimie du Solide Appliquée, Faculté des Sciences, Université Mohammed V-Agdal, Avenue Ibn Battouta, BP 1014, Rabat, Morocco

## Abstract

In the title compound, C_18_H_14_N_4_O_2_, the triazole ring makes dihedral angles of 77.32 (8) and 75.56 (9)°, respectively, with the indoline residue and the terminal phenyl group. In the crystal, mol­ecules are linked by C—H⋯N hydrogen bonds into tapes parallel to the *b* axis. The tapes are linked together by π–π inter­actions between triazole rings [inter-­centroid distance = 3.4945 (9) Å].

## Related literature   

For the biological activity of indoline derivatives, see: Bhrigu *et al.* (2010 ▶); Da Silva *et al.* (2001 ▶); Ramachandran (2011 ▶); Smitha *et al.* (2008 ▶). For structures of indoline-2,3-dione derivatives, see: Qachchachi *et al.* (2013 ▶, 2014 ▶).
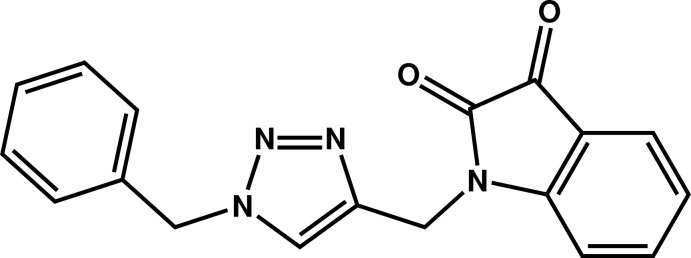


## Experimental   

### 

#### Crystal data  

C_18_H_14_N_4_O_2_
*M* *_r_* = 318.33
Monoclinic, 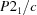

*a* = 11.53860 (18) Å
*b* = 5.38700 (9) Å
*c* = 23.2433 (4) Å
β = 92.1048 (16)°
*V* = 1443.79 (4) Å^3^
*Z* = 4
Cu *K*α radiation
μ = 0.81 mm^−1^
*T* = 123 K
0.20 × 0.04 × 0.02 mm

#### Data collection  

Agilent SuperNova, Single source at offset, Atlas diffractometer
Absorption correction: multi-scan [*CrysAlis PRO* (Agilent, 2013 ▶), using expressions derived from Clark & Reid (1995 ▶)] *T*_min_ = 0.722, *T*_max_ = 1.000
11043 measured reflections
2882 independent reflections
2480 reflections with *I* > 2σ(*I*)
*R*_int_ = 0.032

#### Refinement  

*R*[*F* ^2^ > 2σ(*F* ^2^)] = 0.045
*wR*(*F* ^2^) = 0.123
*S* = 1.04
2882 reflections
217 parameters
H-atom parameters constrained
Δρ_max_ = 0.44 e Å^−3^
Δρ_min_ = −0.22 e Å^−3^

### 

Data collection: *CrysAlis PRO* (Agilent, 2013 ▶); cell refinement: *CrysAlis PRO*; data reduction: *CrysAlis PRO*; program(s) used to solve structure: *SHELXS97* (Sheldrick, 2008 ▶); program(s) used to refine structure: *SHELXL97* (Sheldrick, 2008 ▶); molecular graphics: *ORTEP-3 for Windows* (Farrugia, 2012 ▶); software used to prepare material for publication: *WinGX* (Farrugia, 2012 ▶) and *publCIF* (Westrip, 2010 ▶).

## Supplementary Material

Crystal structure: contains datablock(s) I. DOI: 10.1107/S1600536814008423/bt6975sup1.cif

Structure factors: contains datablock(s) I. DOI: 10.1107/S1600536814008423/bt6975Isup2.hkl

Supporting information file. DOI: 10.1107/S1600536814008423/bt6975Isup3.cml

CCDC reference: 997252

Additional supporting information:  crystallographic information; 3D view; checkCIF report

## Figures and Tables

**Table 1:** Hydrogen-bond geometry (Å, °)

*D*—H⋯*A*	*D*—H	H⋯*A*	*D*⋯*A*	*D*—H⋯*A*
C11—H11⋯N2^i^	0.93	2.50	3.383 (2)	158
C11—H11⋯N3^i^	0.93	2.40	3.313 (2)	167

Symmetry code: (i) 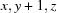.

**Fig. 1.**
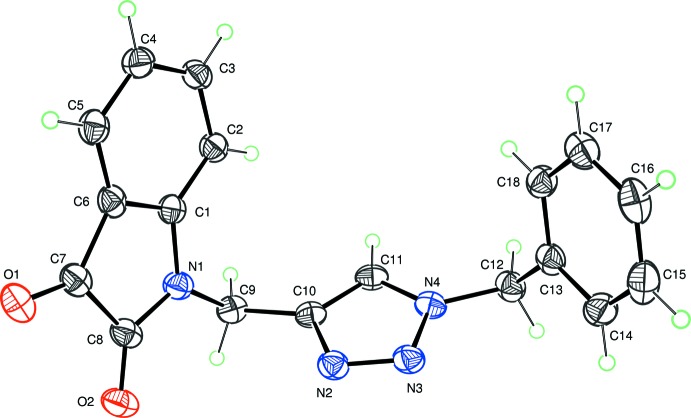
: Molecular plot the title compound with the atom-labelling scheme. Displacement ellipsoids are drawn at the 50% probability level. H atoms are represented as small circles.

**Fig. 2.**
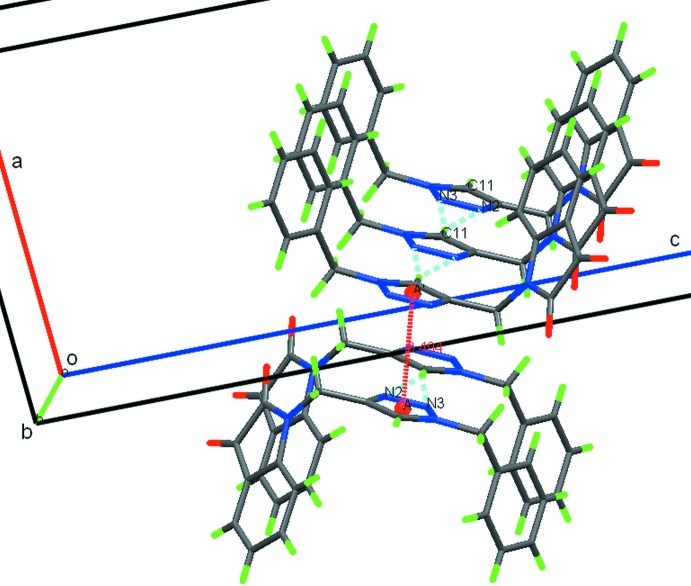
: Intermolecular π–π (red dashed line) and hydrogen interactions (dashed blue lines) in the title compound.

## supplementary crystallographic information

### 1. Comment

Isatin, 1*H*-indole-2,3-dione, is a heterocyclic compound of significant importance in medicinal chemistry. It is a synthetically versatile molecule, a precursor for a large number of pharmacologically active compounds. Isatin and its derivatives have aroused great attention in recent years due to their wide variety of biological activities, relevant to application as insecticides and fungicides and in a broad range of drug therapies, including anticancer drugs, antibiotics and antidepressants (Bhrigu *et al.*, 2010; Da Silva *et al.*, 2001; Ramachandran, 2011; Smitha *et al.*, 2008). As a continuation of our research work devoted to the development of isatin derivatives (Qachchachi *et al.*, 2013, 2014), we report in this paper the synthesis of a new indoline-2,3-dione derivative.

The molecule of title compound is build up from a fused five- and six-membered rings linked to a triazole ring which is connected to a benzyl ring as shown in Fig. 1. The indoline ring and the two carbonyl oxygen atoms are nearly coplanar, with the largest deviation from the mean plane being -0.059 (2) A° at O2 atom. The triazole plane is nearly perpendicular to the mean plane passing through the fused ring system (N1, C1 to C8) and to the terminal phenyl ring (C13 to C18) as indicated by the dihedral angles between them of 77.32 (8)° and 75.56 (9)°, respectively. The indazole system makes a dihedral angle of 77.02 (8)° with the phenyl ring.

In the crystal, the molecules are linked by C11–H11···N2 and C11–H11···N3 hydrogen bonds in the way to build bands parallel to the *b* axis direction. Two bands are linked together by π–π interactions between triazole rings [intercentroid distance = 3.494 Å] as shown in Fig. 2 and Table 1.

### 2. Experimental

To a solution of 1-(prop-2-ynyl)indoline-2,3-dione (0.2 g, 2.4 mmol) dissolved in EtOH/H_2_O (1,1) was added 1-(azidomethyl)benzene (0.4 g, 4.1 mmol), in presence of CuSO_4_. The mixture was stirred for 24 h; the reaction was monitored by thin layer chromatography. The mixture was filtered and the solvent removed under vacuum. The solid obtained was recrystallized from ethanol to afford the title compound as yellow crystals in 81% yield.

### 3. Refinement

All H atoms could be located in a difference Fourier map. Nevertheless, they were placed in calculated positions with C—H = 0.93 Å (aromatic), and C—H = 0.97 Å (methylene) and refined as riding on their parent atoms with *U*_iso_(H) = 1.2 *U*_eq_ (aromatic and methylene).

### Crystal data

**Table:**

C_18_H_14_N_4_O_2_	*F*(000) = 664
*M**_r_* = 318.33	*D*_x_ = 1.464 Mg m^−^^3^
Monoclinic, *P*2_1_/*c*	Cu *K*α radiation, λ = 1.54184 Å
Hall symbol: -P 2ybc	Cell parameters from 4927 reflections
*a* = 11.53860 (18) Å	θ = 3.8–73.3°
*b* = 5.38700 (9) Å	µ = 0.81 mm^−^^1^
*c* = 23.2433 (4) Å	*T* = 123 K
β = 92.1048 (16)°	Rod, clear intense yellow
*V* = 1443.79 (4) Å^3^	0.20 × 0.04 × 0.02 mm
*Z* = 4	

### Data collection

**Table:**

Agilent SuperNova, Single source at offset, Atlas diffractometer	2882 independent reflections
Radiation source: SuperNova (Cu) X-ray Source	2480 reflections with *I* > 2σ(*I*)
Mirror monochromator	*R*_int_ = 0.032
Detector resolution: 10.3546 pixels mm^-1^	θ_max_ = 73.5°, θ_min_ = 3.8°
ω scans	*h* = −14→11
Absorption correction: multi-scan [*CrysAlis PRO* (Agilent, 2013), using expressions derived from Clark & Reid (1995)]	*k* = −6→6
*T*_min_ = 0.722, *T*_max_ = 1.000	*l* = −28→27
11043 measured reflections	

### Refinement

**Table:**

Refinement on *F*^2^	Primary atom site location: structure-invariant direct methods
Least-squares matrix: full	Secondary atom site location: difference Fourier map
*R*[*F*^2^ > 2σ(*F*^2^)] = 0.045	Hydrogen site location: inferred from neighbouring sites
*wR*(*F*^2^) = 0.123	H-atom parameters constrained
*S* = 1.04	*w* = 1/[σ^2^(*F*_o_^2^) + (0.0677*P*)^2^ + 0.6175*P*] where *P* = (*F*_o_^2^ + 2*F*_c_^2^)/3
2882 reflections	(Δ/σ)_max_ = 0.001
217 parameters	Δρ_max_ = 0.44 e Å^−^^3^
0 restraints	Δρ_min_ = −0.22 e Å^−^^3^

### Special details

**Table:**

Geometry. All e.s.d.'s (except the e.s.d. in the dihedral angle between two l.s. planes) are estimated using the full covariance matrix. The cell e.s.d.'s are taken into account individually in the estimation of e.s.d.'s in distances, angles and torsion angles; correlations between e.s.d.'s in cell parameters are only used when they are defined by crystal symmetry. An approximate (isotropic) treatment of cell e.s.d.'s is used for estimating e.s.d.'s involving l.s. planes.
Refinement. Refinement of *F*^2^ against all reflections. The weighted *R*-factor *wR* and goodness of fit *S* are based on *F*^2^, conventional *R*-factors *R* are based on *F*, with *F* set to zero for negative *F*^2^. The threshold expression of *F*^2^ > 2σ(*F*^2^) is used only for calculating *R*-factors(gt) *etc*. and is not relevant to the choice of reflections for refinement. *R*-factors based on *F*^2^ are statistically about twice as large as those based on *F*, and *R*- factors based on all data will be even larger.

### Fractional atomic coordinates and isotropic or equivalent isotropic displacement parameters (Å^2^)

**Table:**

	*x*	*y*	*z*	*U*_iso_*/*U*_eq_	
O1	0.65655 (11)	−0.1406 (2)	1.29231 (5)	0.0327 (3)	
O2	0.48639 (10)	−0.1470 (2)	1.19284 (6)	0.0333 (3)	
N1	0.59263 (12)	0.1979 (3)	1.16704 (6)	0.0258 (3)	
N3	0.66397 (12)	−0.0542 (2)	0.99818 (6)	0.0267 (3)	
N4	0.67538 (12)	0.1811 (3)	0.98001 (6)	0.0255 (3)	
N2	0.61294 (12)	−0.0461 (3)	1.04795 (6)	0.0264 (3)	
C9	0.52982 (14)	0.2675 (3)	1.11417 (7)	0.0269 (4)	
H9A	0.5179	0.4457	1.1140	0.032*	
H9B	0.4541	0.1888	1.1133	0.032*	
C1	0.69237 (13)	0.3209 (3)	1.18873 (7)	0.0235 (3)	
C2	0.74712 (14)	0.5253 (3)	1.16601 (7)	0.0253 (3)	
H2	0.7192	0.6016	1.1324	0.030*	
C4	0.88894 (14)	0.5000 (3)	1.24613 (7)	0.0287 (4)	
H4	0.9552	0.5625	1.2649	0.034*	
C10	0.59134 (13)	0.1956 (3)	1.06114 (7)	0.0242 (3)	
C18	0.94153 (15)	0.2600 (3)	0.94699 (7)	0.0287 (4)	
H18	0.9284	0.4072	0.9666	0.034*	
C6	0.73451 (14)	0.2077 (3)	1.23951 (7)	0.0250 (3)	
C5	0.83290 (14)	0.2949 (3)	1.26844 (7)	0.0276 (4)	
H5	0.8610	0.2184	1.3020	0.033*	
C13	0.84837 (15)	0.1331 (3)	0.92142 (7)	0.0267 (4)	
C3	0.84634 (14)	0.6118 (3)	1.19591 (7)	0.0274 (4)	
H3	0.8851	0.7488	1.1817	0.033*	
C7	0.65586 (14)	0.0030 (3)	1.25238 (7)	0.0264 (4)	
C12	0.72714 (15)	0.2352 (3)	0.92448 (7)	0.0293 (4)	
H12A	0.7291	0.4135	0.9188	0.035*	
H12B	0.6789	0.1639	0.8937	0.035*	
C17	1.05397 (15)	0.1708 (4)	0.94380 (8)	0.0332 (4)	
H17	1.1155	0.2578	0.9611	0.040*	
C16	1.07402 (17)	−0.0482 (4)	0.91471 (8)	0.0356 (4)	
H16	1.1491	−0.1094	0.9125	0.043*	
C11	0.63147 (13)	0.3411 (3)	1.01791 (7)	0.0258 (3)	
H11	0.6289	0.5133	1.0153	0.031*	
C14	0.86952 (17)	−0.0866 (3)	0.89222 (7)	0.0327 (4)	
H14	0.8082	−0.1740	0.8748	0.039*	
C15	0.98220 (18)	−0.1757 (4)	0.88899 (8)	0.0376 (4)	
H15	0.9958	−0.3225	0.8693	0.045*	
C8	0.56440 (14)	0.0017 (3)	1.20128 (7)	0.0268 (4)	

### Atomic displacement parameters (Å^2^)

**Table:**

	*U*^11^	*U*^22^	*U*^33^	*U*^12^	*U*^13^	*U*^23^
O1	0.0334 (6)	0.0294 (6)	0.0359 (7)	0.0032 (5)	0.0104 (5)	0.0061 (5)
O2	0.0274 (6)	0.0285 (6)	0.0444 (7)	−0.0058 (5)	0.0064 (5)	−0.0018 (5)
N1	0.0235 (6)	0.0253 (7)	0.0286 (7)	−0.0017 (5)	0.0023 (5)	−0.0016 (5)
N3	0.0303 (7)	0.0193 (7)	0.0307 (7)	−0.0013 (5)	0.0024 (6)	0.0006 (5)
N4	0.0250 (7)	0.0215 (7)	0.0300 (7)	−0.0017 (5)	−0.0001 (5)	0.0026 (5)
N2	0.0275 (7)	0.0220 (7)	0.0299 (7)	−0.0005 (5)	0.0026 (5)	0.0003 (5)
C9	0.0239 (7)	0.0259 (8)	0.0307 (8)	0.0012 (6)	0.0000 (6)	−0.0031 (6)
C1	0.0223 (7)	0.0236 (8)	0.0250 (7)	0.0021 (6)	0.0054 (6)	−0.0039 (6)
C2	0.0269 (8)	0.0234 (8)	0.0259 (8)	0.0001 (6)	0.0040 (6)	−0.0015 (6)
C4	0.0254 (8)	0.0318 (9)	0.0288 (8)	−0.0024 (7)	0.0018 (6)	−0.0049 (7)
C10	0.0201 (7)	0.0225 (8)	0.0298 (8)	0.0008 (6)	−0.0027 (6)	−0.0019 (6)
C18	0.0337 (9)	0.0257 (8)	0.0267 (8)	−0.0003 (7)	0.0032 (7)	−0.0006 (6)
C6	0.0256 (7)	0.0242 (8)	0.0256 (8)	0.0027 (6)	0.0076 (6)	−0.0017 (6)
C5	0.0270 (8)	0.0306 (8)	0.0255 (8)	0.0032 (6)	0.0039 (6)	−0.0014 (6)
C13	0.0323 (8)	0.0242 (8)	0.0238 (7)	−0.0008 (6)	0.0033 (6)	0.0061 (6)
C3	0.0267 (8)	0.0256 (8)	0.0303 (8)	−0.0030 (6)	0.0075 (6)	−0.0025 (6)
C7	0.0267 (8)	0.0219 (8)	0.0310 (8)	0.0030 (6)	0.0090 (6)	−0.0011 (6)
C12	0.0320 (8)	0.0292 (9)	0.0268 (8)	−0.0017 (7)	0.0010 (6)	0.0056 (7)
C17	0.0304 (9)	0.0377 (10)	0.0315 (9)	−0.0009 (7)	0.0032 (7)	0.0051 (7)
C16	0.0377 (9)	0.0379 (10)	0.0318 (9)	0.0098 (8)	0.0108 (7)	0.0092 (8)
C11	0.0239 (8)	0.0190 (7)	0.0343 (9)	0.0004 (6)	−0.0029 (6)	−0.0008 (6)
C14	0.0430 (10)	0.0266 (9)	0.0284 (8)	−0.0052 (7)	0.0022 (7)	0.0005 (7)
C15	0.0555 (12)	0.0277 (9)	0.0305 (9)	0.0063 (8)	0.0130 (8)	0.0023 (7)
C8	0.0238 (8)	0.0227 (8)	0.0343 (9)	0.0001 (6)	0.0081 (6)	−0.0024 (6)

### Geometric parameters (Å, º)

**Table:**

O1—C7	1.208 (2)	C18—C17	1.388 (3)
O2—C8	1.216 (2)	C18—C13	1.389 (2)
N1—C8	1.370 (2)	C18—H18	0.9300
N1—C1	1.406 (2)	C6—C5	1.380 (2)
N1—C9	1.453 (2)	C6—C7	1.466 (2)
N3—N2	1.318 (2)	C5—H5	0.9300
N3—N4	1.3436 (19)	C13—C14	1.390 (2)
N4—C11	1.345 (2)	C13—C12	1.507 (2)
N4—C12	1.471 (2)	C3—H3	0.9300
N2—C10	1.362 (2)	C7—C8	1.560 (2)
C9—C10	1.496 (2)	C12—H12A	0.9700
C9—H9A	0.9700	C12—H12B	0.9700
C9—H9B	0.9700	C17—C16	1.383 (3)
C1—C2	1.384 (2)	C17—H17	0.9300
C1—C6	1.400 (2)	C16—C15	1.380 (3)
C2—C3	1.397 (2)	C16—H16	0.9300
C2—H2	0.9300	C11—H11	0.9300
C4—C3	1.387 (2)	C14—C15	1.391 (3)
C4—C5	1.390 (2)	C14—H14	0.9300
C4—H4	0.9300	C15—H15	0.9300
C10—C11	1.369 (2)		
			
C8—N1—C1	111.37 (14)	C4—C5—H5	120.8
C8—N1—C9	124.67 (14)	C18—C13—C14	118.74 (16)
C1—N1—C9	123.94 (14)	C18—C13—C12	120.34 (16)
N2—N3—N4	107.26 (13)	C14—C13—C12	120.91 (16)
N3—N4—C11	110.80 (13)	C4—C3—C2	122.16 (16)
N3—N4—C12	120.69 (14)	C4—C3—H3	118.9
C11—N4—C12	128.48 (14)	C2—C3—H3	118.9
N3—N2—C10	108.69 (13)	O1—C7—C6	130.68 (17)
N1—C9—C10	113.14 (13)	O1—C7—C8	124.58 (15)
N1—C9—H9A	109.0	C6—C7—C8	104.74 (13)
C10—C9—H9A	109.0	N4—C12—C13	112.11 (13)
N1—C9—H9B	109.0	N4—C12—H12A	109.2
C10—C9—H9B	109.0	C13—C12—H12A	109.2
H9A—C9—H9B	107.8	N4—C12—H12B	109.2
C2—C1—C6	121.28 (15)	C13—C12—H12B	109.2
C2—C1—N1	128.14 (15)	H12A—C12—H12B	107.9
C6—C1—N1	110.58 (14)	C16—C17—C18	119.66 (18)
C1—C2—C3	116.92 (15)	C16—C17—H17	120.2
C1—C2—H2	121.5	C18—C17—H17	120.2
C3—C2—H2	121.5	C15—C16—C17	119.75 (17)
C3—C4—C5	120.24 (16)	C15—C16—H16	120.1
C3—C4—H4	119.9	C17—C16—H16	120.1
C5—C4—H4	119.9	N4—C11—C10	105.03 (14)
N2—C10—C11	108.21 (14)	N4—C11—H11	127.5
N2—C10—C9	121.95 (15)	C10—C11—H11	127.5
C11—C10—C9	129.80 (15)	C13—C14—C15	120.10 (17)
C17—C18—C13	121.12 (17)	C13—C14—H14	120.0
C17—C18—H18	119.4	C15—C14—H14	120.0
C13—C18—H18	119.4	C16—C15—C14	120.62 (17)
C5—C6—C1	121.09 (16)	C16—C15—H15	119.7
C5—C6—C7	131.36 (16)	C14—C15—H15	119.7
C1—C6—C7	107.54 (15)	O2—C8—N1	127.25 (16)
C6—C5—C4	118.32 (16)	O2—C8—C7	127.03 (16)
C6—C5—H5	120.8	N1—C8—C7	105.70 (13)

### Hydrogen-bond geometry (Å, º)

**Table:**

*D*—H···*A*	*D*—H	H···*A*	*D*···*A*	*D*—H···*A*
C11—H11···N2^i^	0.93	2.50	3.383 (2)	158
C11—H11···N3^i^	0.93	2.40	3.313 (2)	167

Symmetry code: (i) *x*, *y*+1, *z*.
